# Epigenetic age acceleration enhances prediction of overall survival in early-stage non-small cell lung cancer

**DOI:** 10.1016/j.isci.2026.117843

**Published:** 2026-10-16

**Authors:** Isabella Gandara, Mulong Du, Yi Li, Li Su, Jui Kothari, Michael Lanuti, Justin F. Gainor, David C. Christiani

**Affiliations:** 1 Department of Biological Engineering, Massachusetts Institute of Technology, Cambridge MA, USA; 2 Department of Environmental Health, Harvard T.H. Chan School of Public Health, Boston, MA, USA; 3 Department of Biostatistics, Center for Global Health, School of Public Health, Nanjing Medical University, Nanjing, China; 4 Department of Biostatistics, University of Michigan School of Public Health, Ann Arbor, MI, USA; 5 Department of Medicine, Pulmonary and Critical Care Division, Massachusetts General Hospital and Harvard Medical School, Boston, MA, USA; 6 Division of Thoracic Surgery, Massachusetts General Hospital and Harvard Medical School, Boston, MA, USA; 7 Massachusetts General Hospital Cancer Center and Department of Hematology & Oncology, Massachusetts General Hospital and Harvard Medical School, Boston, MA, USA

**Keywords:** aging, prognosis, biomarkers, epigenomics, survival, thoracic oncology

## Abstract

Epigenetic age, influenced by environmental factors like smoking, predicts mortality in general populations but remains under-explored in early-stage lung cancer. We analyzed blood DNA methylation from 75 Boston Lung Cancer Study participants with early-stage non-small cell lung cancer (NSCLC), of whom 63 died during follow-up. Epigenetic age acceleration (EAA) was estimated using Horvath, Hannum, PhenoAge, and GrimAge2 clocks and assessed using Cox models and random survival forests (RSFs). Across clocks, EAA significantly predicted survival in early-stage NSCLC. GrimAge2 EAA achieved the highest predictive accuracy (C index = 0.73±0.04) and emerged as the dominant predictor of survival among RSF models (variable importance = 5.7%), surpassing smoking and age. GrimAge2 EAA showed a non-linear association with mortality in which current smokers had >10% higher predicted mortality risk than former or never smokers at equivalent EAA, a pattern confirmed in Kaplan-Meier analyses. GrimAge2 EAA is a promising biomarker for personalizing risk stratification alongside traditional clinical factors.

## Highlights

• Epigenetic age acceleration enhances survival prediction beyond traditional risk factors
• GrimAge2 shows strongest discrimination across Cox and random survival forest models
• Current smokers experience higher mortality across GrimAge2 acceleration levels

## Graphical abstract

**Figure:**
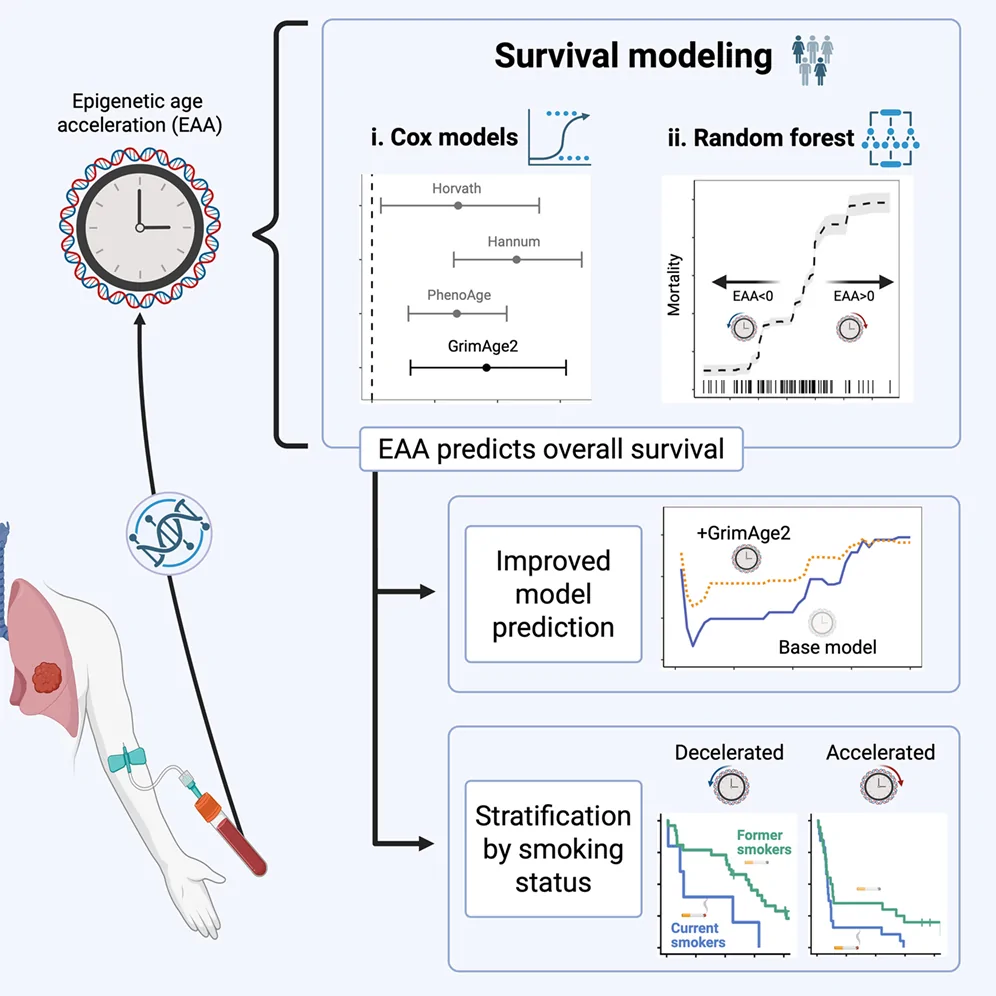

## Introduction

Lung cancer, particularly non-small cell lung cancer (NSCLC), remains the leading cause of cancer-related deaths in the United States, accounting for approximately 20% of all cancer fatalities.^1^ In 2025, an estimated 226,650 new cases of lung cancer are projected to be diagnosed, with 124,730 deaths attributed to the disease.^1^ Although advances in early detection and targeted therapies have improved survival rates, outcomes for NSCLC patients remain highly variable.^2^ Nearly one-third of NSCLC cases in the United States are diagnosed at an early stage^3^; however, survival rates for early-stage NSCLC are significantly lower than those of other common cancers. In comparison to localized breast and prostate cancers, the two most diagnosed malignancies in the United States, whose 5-year survival rates each exceed 99%,^1^ the 5-year relative survival rate for localized NSCLC is only about 68%.^3^ This disparity demonstrates the urgent need to identify biomarkers that can capture the heterogeneity in NSCLC outcomes, improving prognosis prediction and allowing for more informed clinical decision making.

Epigenetic age, estimated through DNA methylation data, has emerged as a promising biomarker of biological aging and disease susceptibility.^4^ Unlike chronological age, which provides a measure of time elapsed since birth, epigenetic age reflects cumulative molecular changes influenced by genetic, environmental, and lifestyle factors.^5^ Epigenetic clocks can be further divided into “first generation clocks” such as the Horvath and Hannum clocks whose designs have focused on predicting chronological age through DNA methylation, while later generation clocks such as the PhenoAge and GrimAge clocks have been developed that incorporate methylation-based surrogates of clinical biomarkers such as C-reactive protein and blood glucose to capture biological signals associated with aging.^6^ Deviations from chronological age, termed epigenetic age acceleration (EAA), have been associated with a range of adverse health outcomes including cancer incidence, cardiovascular disease, and all-cause mortality in a variety of epigenetic clock studies.^5^^,^^7^^,^^8^^,^^9^

In lung cancer, EAA may provide unique insights into patient prognosis because it captures the impact of both environmental exposures and biological factors. While smoking remains a dominant risk factor for lung cancer, exposures such as air pollution, occupational carcinogens, and secondhand smoke are also significant contributors, particularly for the growing subset of never-smokers diagnosed with lung cancer.^10^^,^^11^ Consequently, EAA may offer a more comprehensive assessment of mortality and morbidity risk compared to traditional metrics, such as smoking history or chronological age, by capturing systemic factors associated with risk, such as carcinogenesis.^12^

Although epigenetic clocks have been studied previously in the context of their ability to predict lung cancer incidence, their role in prediction of mortality in lung cancer remains under-explored. This study aims to address this gap by investigating whether EAA predicts overall survival in a sample of 75 early-stage NSCLC patients, and whether EAA provides prognostic value beyond established risk factors. Using Cox proportional hazards models and random survival forests (RSFs), we evaluate the predictive performance of four epigenetic clocks and examine whether associations between EAA and mortality differ by smoking status, with implications for personalized risk stratification in early-stage lung cancer.

## Results

### Study population and baseline characteristics

Epigenetic age was calculated for 75 individuals diagnosed with early-stage NSCLC from 1998 to 2008 using blood methylation data collected at diagnosis (Table 1), for four clocks: Horvath, Hannum, PhenoAge, and GrimAge2. All participants were treated by surgical resection with curative intent; 8 participants (10.7%) also received adjuvant therapy following surgery, including chemotherapy (*n* = 4, 5.3%), radiotherapy (*n* = 3, 4.0%), or both (*n* = 1, 1.3%). The study sample had a mean chronological age of 69.4 years (SD = 8.2), with most participants being either former smokers (*n* = 46, 61.3%) or current smokers (*n* = 24, 32.0%). Lung adenocarcinoma (LUAD) was the most common histological subtype (*n* = 29, 38.7%), followed by squamous cell carcinoma (LUSC; *n* = 18, 24%) and bronchioalveolar carcinoma, which has since been reclassified to adenocarcinoma *in situ* (BAC; *n* = 15, 20%). The remaining 17.3% (*n* = 13) included large cell carcinoma (*n* = 4), mixed histology (*n* = 3), multiple primary tumors (*n* = 1), and unspecified NSCLC (*n* = 5). Clinical stage was balanced between IA (*n* = 38, 50.7%) and IB (*n* = 36, 48.0%), with one case classified as stage I only. During follow-up, 63 deaths (84.0%) and 10 relapses (13.3%) occurred.

**Table 1:** Baseline characteristics

Characteristic	Overall (*N* = 75)	Short-term (*n* = 39)	Long-term (*n* = 36)	*p* value^a^
Age, years	69.4 (8.2)	70.5 (8.2)	68.3 (8.1)	0.26
Male sex	43 (57.3%)	23 (59.0%)	20 (55.6%)	0.82
Smoking status				0.002
Never smoker	5 (6.7%)	1 (2.6%)	4 (11.1%)	–
Former Smoker	46 (61.3%)	19 (48.7%)	27 (75.0%)	–
Current Smoker	24 (32.0%)	19 (48.7%)	5 (13.9%)	–
Pack-years	43.0 (24.0−69.7)	49.2 (30.0−77.3)	34.0 (20.3−53.4)	0.02
EAA, years				–
Horvath Clock	0.0 (6.1)	1.0 (7.0)	−1.1 (4.7)	0.13
Hannum Clock	0.0 (5.5)	1.4 (6.0)	−1.5 (4.4)	0.02
PhenoAge Clock	0.0 (7.7)	1.7 (8.3)	−1.8 (6.6)	0.05
GrimAge2 Clock	0.0 (7.3)	3.4 (6.9)	−3.7 (5.9)	<0.001
Clinical Stage				0.36
I	1 (1.3%)	0 (0%)	1 (2.8%)	–
IA	38 (50.7%)	18 (46.2%)	20 (55.6%)	–
IB	36 (48.0%)	21 (53.8%)	15 (41.7%)	–
Histologic subtype				0.09
LUAD	29 (38.7%)	13 (33.3%)	16 (44.4%)	–
BAC	15 (20.0%)	5 (12.8%)	10 (27.8%)	–
LUSC	18 (24.0%)	11 (28.2%)	7 (19.4%)	–
Other	13 (17.3%)	10 (25.6%)	3 (8.3%)	–
Treatment				0.71
Surgery only	67 (89.3%)	34 (87.2%)	33 (91.7%)	–
Surgery + Adjuvant therapy^b^	8 (10.7%)	5 (12.8%)	3 (8.3%)	–
Relapse	10 (13.3%)	2 (5.1%)	8 (22.2%)	–
Death	63 (84.0%)	39 (100%)	24 (66.7%)	–
Follow-up time, months	34.1 (19.5−186.2)	19.9 (13.7−28.1)	187.7 (144.8−244.8)	–
Time to death, months	28.9 (18.3−151.7)	19.9 (13.7−28.1)	169.2 (133.5−192.2)	–

Continuous variables are shown as mean (SD), or median (IQR) while categorical variables are shown as *n* (%).

a *p* values for continuous baseline predictors were calculated using *t* test or Wilcoxon rank sum test, and with Fisher’s exact test for categorical baseline data.

b Adjuvant therapy includes chemotherapy (*n* = 4), radiotherapy (*n* = 3), or both (*n* = 1).

Compared to long-term survivors (*n* = 36), short-term survivors (*n* = 39) were more likely to be current smokers and have stage 1B disease, but less likely to have LUAD or BAC histology. Short-term survivors also had higher pack-years and were slightly older and more likely to be male. Among those receiving adjuvant therapy, 5 were short-term survivors (3 chemotherapy, 2 radiotherapy), and 3 were long-term survivors (1 chemotherapy, 1 radiotherapy, 1 both). Mean EAA (defined as the residual of epigenetic age regressed on chronological age) was consistently positive among short-term survivors and negative among long-term survivors across all clocks. The largest difference was observed for the GrimAge2 clock, where short-term survivors showed a mean acceleration of +3.4 years (SD = 6.9), while long-term survivors had a mean EAA of –3.7 years (SD = 5.9).

### Epigenetic age acceleration predicts overall survival across clocks

We employed Cox proportional hazards models^13^ to assess the effect of EAA on overall survival, applying inverse probability of selection weighting (IPSW) to account for the extreme phenotype sampling design. IPSW adjustment accounts for the different sampling fractions of short- and long-term survivors, representing a weighted pseudopopulation that approximates the distribution of extreme survivors in the eligible BLCS cohort, as detailed in methods. Due to limited sample size, we limited our adjustment set to age, sex, and smoking status for primary analysis.

All epigenetic clocks showed significant associations with mortality (Figure 1). Hannum EAA demonstrated the strongest hazard ratio (HR = 1.09, 95% CI:1.05–1.13, *p* = 3.64 × 10^-6^), while GrimAge2 EAA showed marginally higher predictive discrimination, as indicated by C index (0.73 vs 0.72 for Hannum; Table 2). Horvath EAA showed the weakest association (HR = 1.05, 95% CI: 1.01–1.11, *p* = 0.029) with a small decrease in C index over the base model (ΔC = –0.005).

**Figure 1:**
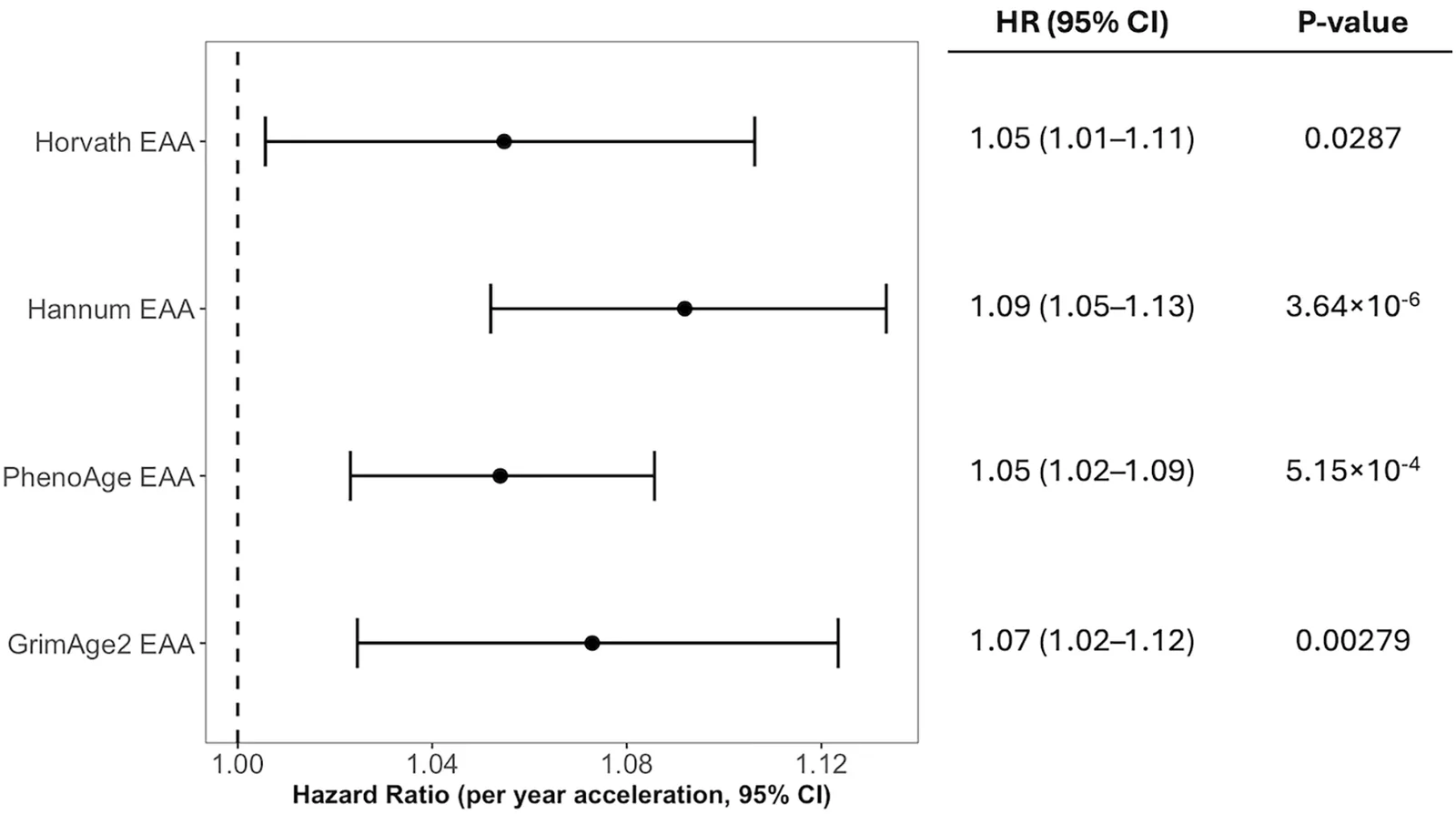
EAA predicts mortality Forest plot showing HRs, 95% confidence intervals, and *p* values for EAA across four clocks. All Cox models are IPSW-weighted and adjusted for chronological age, sex, and smoking status. HRs represent mortality risk per one year increase in EAA.

**Table 2:** Model performance comparison

Model	HR (95% CI)	C index (SE)	RSF Error (SD)	BCVI (SD)
Base	–	0.69 (0.04)	38.4% (0.4)	–
Pack-years	–	0.70 (0.04)	37.9% (0.4)	2.0% (0.3)
Horvath EAA	1.05 (1.01–1.11)	0.69 (0.04)	36.3% (0.4)	1.4% (0.2)
Hannum EAA	1.09 (1.05–1.13)	0.72 (0.04)	36.1% (0.3)	1.7% (0.2)
PhenoAge EAA	1.05 (1.02–1.09)	0.71 (0.04)	36.3% (0.4)	2.6% (0.3)
GrimAge2 EAA	1.07 (1.02–1.12)	0.73 (0.03)	33.6% (0.4)	5.7% (0.4)

IPSW-weighted models adjusted for age, sex, and smoking status. HR refers to hazard ratio per year of EAA, or per unit log_10_ pack-years. For RSF metrics, the mean (SD) from 500 iterations is reported. Error rate is the out-of-bag error rate and BCVI is the Breiman-Cutler variable importance.

Given the known associations of smoking with accelerated epigenetic aging,^5^^,^^7^^,^^14^ we also compared EAA models to a pack-years model, using log_10_-transformed pack-years as a reference predictor while adjusting for age, sex, and smoking status to assess whether EAA provides superior predictive value compared to self-reported cumulative smoking exposure. With the exception of Horvath, all EAA models showed superior predictive discrimination compared to the pack-years model. For example, GrimAge2 improved C index by +0.042 over the base model, compared to +0.008 for pack-years alone.

Because smoking status was a significant predictor in all Cox models (all *p* < 0.05), we also tested for effect modification by smoking status using interaction models. In each case, the Rao-Scott likelihood ratio test for interaction models was nonsignificant (all *p* >0.1), indicating that the association between mortality and EAA does not differ significantly by smoking group.

To assess predictive performance over time, time-dependent AUCs were computed in the analytic sample up to 20 years post-diagnosis (Figure 2A). Due to the extreme survival selection sampling described in methods, a gap in mortality data exists between 2.9 and 7.6 years; however, consistent patterns in time-dependent AUC were observed for both short- and long-term survivors. All EAA models except Horvath outperformed the base and pack-years models across time points through 14 years. GrimAge2 EAA consistently showed the highest AUC (range: 0.73–0.86 for 0–14 years), followed by Hannum and PhenoAge, with comparable performance between models. After 14 years, discrimination metrics converged as number at risk decreased (*n* = 24).

**Figure 2:**
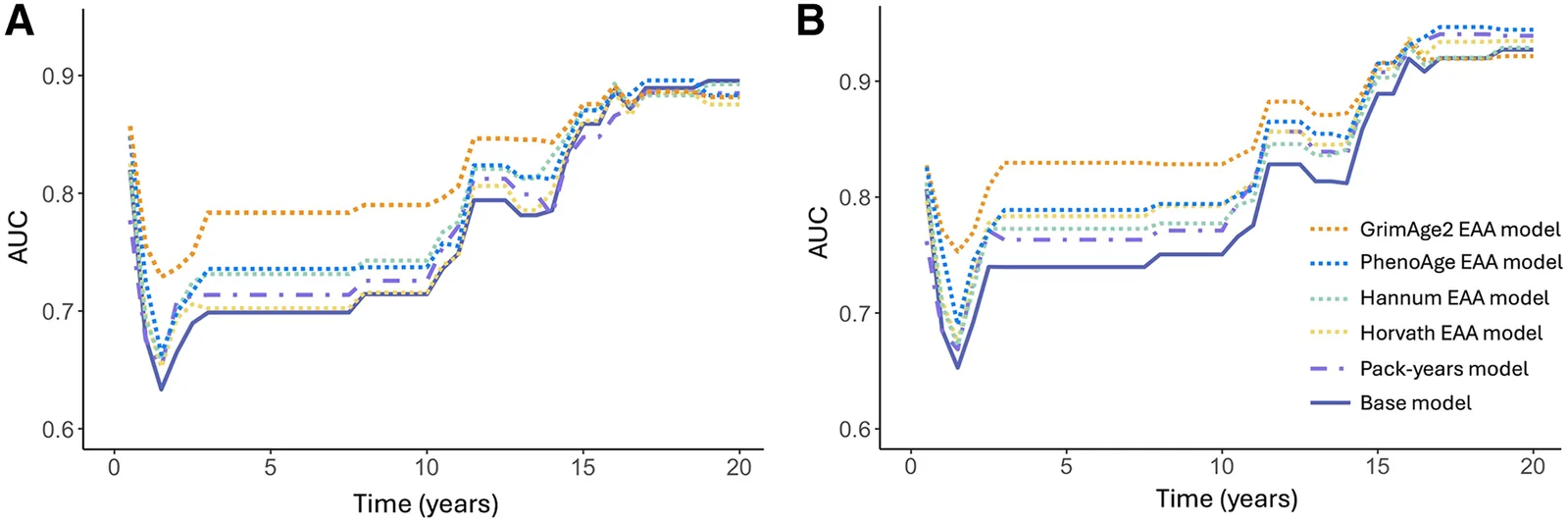
Time-dependent AUC of Cox and RSF models (A) Time-dependent AUC for Cox models of overall survival, where time refers to time after diagnosis. A separate pack-years model was constructed as a reference, incorporating log_10_-transformed pack-years as an added covariate to the base model. (B) Time-dependent AUC for RSF models. The mean AUC from 500 iterations is reported to account for randomness.

### Random survival forests confirm GrimAge2 as an important predictor of mortality

RSF models^15^ with IPSW-based sampling probabilities were employed to investigate the effects of EAA on mortality risk with non-parametric modeling. RSF models, which are an extension of the random forest ensemble learning method for time-to-event data, are particularly useful in gaining insight into survival data compared to traditional Cox models due to their capacity to rank feature importance and provide insights into the relationship between individual variables and mortality risk through partial dependence plots.^16^^,^^17^^,^^18^ To account for variability between random forests and obtain robust estimates, we refit each RSF model 500 times and report mean performance metrics with standard deviation.

GrimAge2 EAA consistently delivered superior predictive performance, with the lowest out-of-bag prediction error (33.6%, SD = 0.4) compared to the base model (38.4%, SD = 0.4). Time-dependent AUC from RSF models (Figure 2B) confirmed the enhanced predictive discrimination of GrimAge2 through 14 years, followed by the Horvath and PhenoAge models.

RSF also offers a feature importance metric known as the Breiman-Cutler variable importance (BCVI), which quantifies the contribution of each variable to the model’s predictive performance based on the loss of predictive capacity on out-of-bag observations when the variable’s values are randomly permuted.^19^ A larger feature importance thus corresponds to a larger drop in accuracy when the feature, or predictor variable, is shuffled. For the base model, smoking status emerged as the most important variable, with a BCVI of 7.4% (SD = 0.3), followed by chronological age (BCVI = 1.8%, SD = 0.3). In the Horvath, Hannum, and PhenoAge models, smoking remained dominant (BCVI = 6.2%, 5.8%, and 5.8%, respectively), representing the most important predictor in 100% of 500 iterations across all three models; in these models, EAA showed modest importance (1.4%, 1.7%, and 2.6%). By contrast, in the GrimAge2 model, GrimAge2 EAA was the most important predictor (BCVI = 5.7%, SD = 0.4) in 97.6% of iterations, surpassing smoking status (4.7%, SD = 0.3) and age (1.2%, SD = 0.2).

### EAA associations remain robust after adjusting for pack-years and clinical factors

To determine whether EAA provides prognostic information beyond self-reported cumulative smoking exposure, we fit models adjusted for both EAA and pack-years (Table S2). As expected, GrimAge2 EAA, which includes a methylation-based proxy for pack-years and demonstrated the highest correlation with pack-years (Pearson r = 0.31, R^2^ = 0.096, Figure S2), showed the greatest attenuation (HR: 1.06 vs. 1.07 in primary model, –1.2% change), though change was minimal and GrimAge2 remained statistically significant (*p* = 0.023). Other clocks showed similarly modest changes (PhenoAge: –0.8%; Hannum: –1.0%; Horvath: +0.1%). Importantly, all pack-years adjusted EAA models improved discrimination beyond pack-years alone. GrimAge2 produced the greatest improvement in both Cox models (ΔC-index = +0.028 from pack-years only model) and RSF models (ΔError rate: –3.8%). GrimAge2 EAA additionally remained the most important predictor by variable importance ranking (BCVI = 4.7%, SD = 0.3) in the GrimAge model even after pack-years adjustment.

We also assessed robustness to adjustment for clinical stage and histologic subtype, finding minimal attenuation in HRs across all clocks; further adjustment for adjuvant therapy yielded similar results (Table S2). For GrimAge2, the HR changed by only +0.1% (HR = 1.07, 95% CI: 1.02–1.13, *p* = 0.007). EAA also improved prediction over clinical covariates alone in both Cox and RSF models. Stage- and histology-adjusted Hannum, PhenoAge, and GrimAge2 models showed comparable discrimination in Cox models (all C index = 0.74). In RSF models, reductions in error rates were also similar across clocks, though GrimAge2 achieved the greatest reduction and remained the only EAA metric ranked as the most important predictor (BCVI = 4.3%, SD = 0.3), as with the pack-years model.

### Mortality risk gradients for GrimAge2 EAA differ by smoking status

To explore the association between GrimAge2 EAA and mortality, partial dependence plots were generated using the GrimAge2 EAA RSF model, revealing a non-linear relationship (Figure 3A). For positive EAA values, or accelerated aging, predicted mortality risk increased approximately linearly from 39% at 0 years acceleration to 50% at +5 years. Beyond 5 years of EAA, mortality risk plateaued before increasing slightly to 55% beyond 11 years acceleration. For negative EAA values, or decelerated aging, the effect was more modest; mortality risk remained stable at 39% for EAA values between 0 and −4 years, decreased to 34% at EAA of –6 years, then plateaued at 33% for greater deceleration.

**Figure 3:**
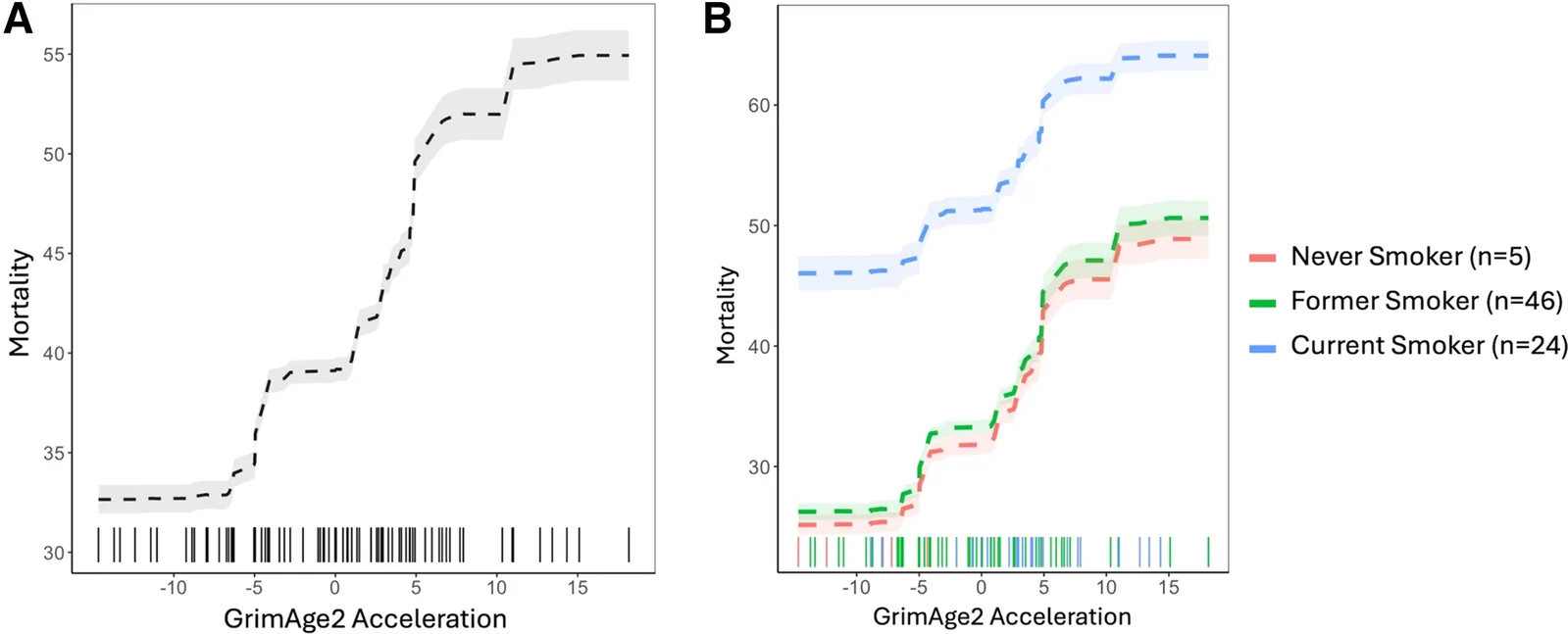
Partial dependence of mortality on GrimAge2 EAA (A) Partial dependence plot showing predicted mortality of as a function of GrimAge2 EAA from the IPSW-weighted RSF model. Tick marks along the *x* axis represent individual observations of EAA values in the sample. The bold dashed line indicates the mean estimated effect of GrimAge2 EAA on mortality using out-of-bag predictions, while shaded region shows ± 2 SD from 500 iterations. (B) Two-way partial dependence plot of mortality on GrimAge2 EAA and smoking status. Colored dashed lines represent the mean EAA-mortality relationship ± 2 SD for smoking status subgroups, while tick mark colors represent the smoking status of individuals in the sample.

When stratified by smoking status (Figure 3B), we observed a consistent shape in the relationship between GrimAge2 EAA and mortality risk across all smoking groups, consistent with the absence of statistical interaction in Cox models. However, absolute mortality risk differed substantially by smoking status. Former smokers showed predicted mortality risks similar to never smokers at equivalent EAA values. For example, GrimAge2 EAA below −10 years was associated with mortality risk of 25% in never smokers and 26% in former smokers, although this comparison is limited by the low number of never smokers in the sample (*n* = 5) and the fact that no never-smokers had positive GrimAge2 EAA values. The most pronounced difference was observed between current and former smokers: at all levels of GrimAge2 EAA, current smokers demonstrated markedly higher mortality risk than former smokers, such as 47% mortality at EAA of −5 years in current smokers, compared to only 28% in former smokers; for EAA of +5 years, current smokers had a predicted mortality risk of 61% compared to 45% in former smokers.

### GrimAge2 acceleration defines distinct patterns of survival

To further characterize the prognostic utility of EAA, we estimated IPSW-standardized Kaplan-Meier survival curves to groups defined by GrimAge2 acceleration status. Individuals with positive GrimAge2 EAA values were classified as *Accelerated*, and those with negative values as *Decelerated* (Figure 4A). IPSW-weighted effective numbers at risk (pseudocounts) and unweighted numbers at risk are detailed in Table S3. Differences between groups were assessed using an IPSW-weighted log rank test. Significant differences were observed across groups defined by GrimAge2 EAA (*p* = 0.0008) while neither Horvath, Hannum, nor PhenoAge EAA resulted in significant differences between accelerated and decelerated individuals (Horvath *p* = 0.83; Hannum *p* = 0.30; PhenoAge *p* = 0.13).

**Figure 4:**
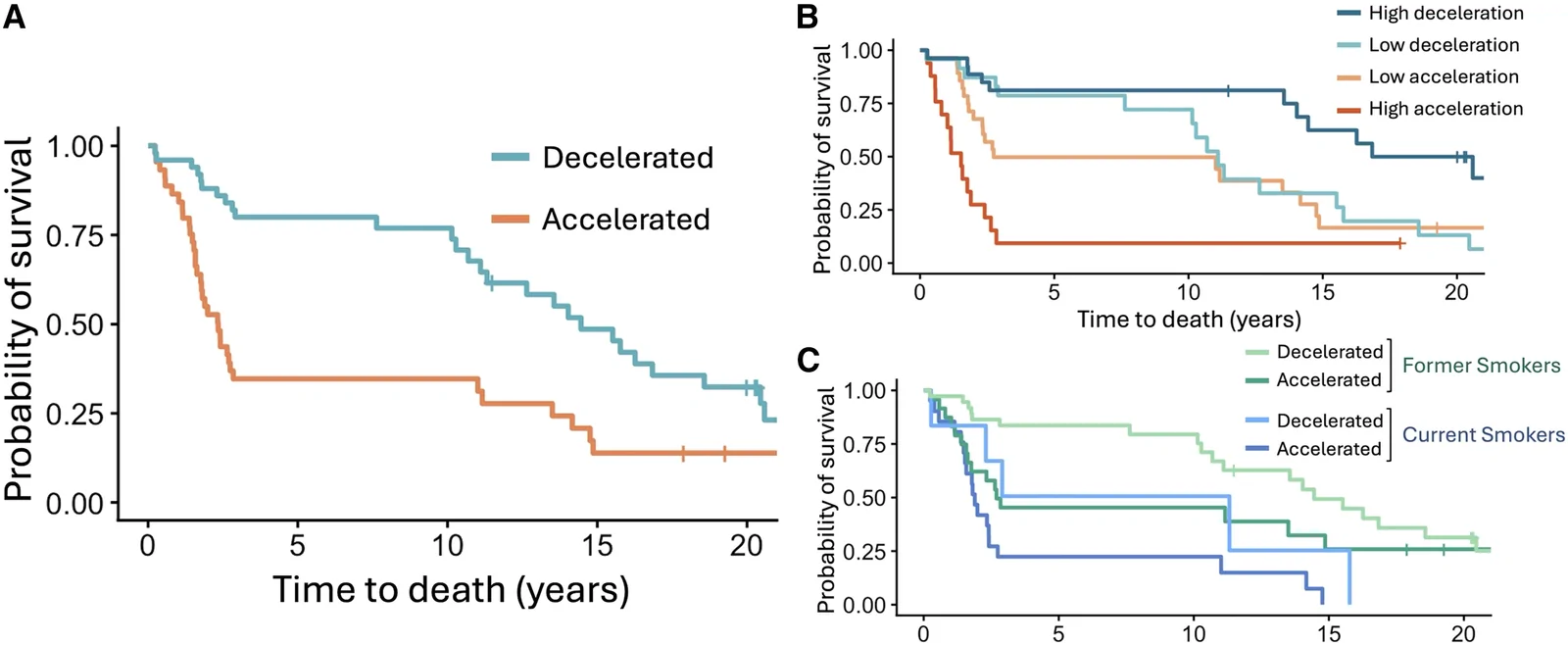
IPSW-weighted Kaplan-Meier survival curves stratified by GrimAge2 EAA (A) Survival for accelerated (EAA > 0) vs. decelerated (EAA ≤ 0) groups. (B) Survival stratified by EAA level, with thresholds defined from plateau in mortality risk (Figure 3A). (C) Survival for accelerated vs. decelerated groups, stratified by smoking status. Never smokers were excluded due to small sample size.

Based on observations from the partial dependence plots, we defined two benchmarks to identify extreme epigenetic aging effects, defined by the plateauing of mortality risk: *high acceleration*, defined as those with GrimAge2 EAA of greater than +5 years, and *high deceleration*, defined as those with GrimAge2 EAA of less than −6 years (Table S4). These subgroups showed strong differences in survival curves over time (Figure 4B). In the analytic sample, 94% (*n* = 15) of the 16 individuals in the *high acceleration* group died within 5 years, compared to only 26% (*n* = 5) of the 19 individuals in the *high deceleration* group; in IPSW-weighted analyses, differences between groups resulted in highly significant differences in survival (*p* = 2.61 × 10^–4^). Moderate levels of acceleration and deceleration were also associated with disparities in survival, although much less pronounced. Consistent with the narrower range of mortality risk associated with negative EAA values in the partial dependence plot, differences between *low* and *high deceleration* groups were smaller than between acceleration groups, particularly in the first 10 years.

Because mortality varied by smoking status, we applied acceleration and deceleration categories to smoking status to compare four groups: accelerated former smokers, decelerated former smokers, accelerated current smokers, and decelerated current smokers (Figure 4C; Table S5). Never smokers were excluded due to low representation in the sample. The resulting IPSW-adjusted Kaplan-Meier plot revealed marked differences between these groups. Decelerated former smokers had the highest probability of survival across timepoints, while accelerated current smokers had the lowest probability of survival, with a probability of only 16% at 3 years post-diagnosis in the analytic sample and no survivors beyond 15 years post-diagnosis. Interestingly, accelerated former smokers and decelerated current smokers demonstrated similar survival probabilities, though the low number of decelerated current smokers (*n* = 5) warrants caution in further interpretation. Despite small subgroup sizes, differences in IPSW-adjusted survival curves for current and former smokers were statistically significant within both subsets of accelerated individuals (*p* = 0.008, *n* = 39) and decelerated individuals (*p* = 0.020, *n* = 31), further indicating the distinct probabilities of survival shaped by both EAA and smoking status.

## Discussion

Our results indicate that applying EAA improves the predictive ability of overall survival models in early-stage NSCLC, with GrimAge2 emerging as the most powerful predictor across both Cox proportional hazards and RSF models. While Hannum EAA was associated with the strongest HR in Cox models, GrimAge2 consistently ranked as the most important variable in RSFs, and demonstrated superior predictive discrimination through 14 years post-diagnosis across both Cox and RSF models.

These findings align with prior research implicating EAA, particularly GrimAge, in predicting diverse health outcomes, including cancer, cardiovascular disease, diabetes, and depression.^5^^,^^7^^,^^9^^,^^20^ The design of GrimAge2 may explain its superior performance to other clocks, including PhenoAge. As a second-generation epigenetic clock, GrimAge2 was explicitly developed to predict mortality using DNA methylation-based surrogates for clinical biomarkers such as C-reactive protein, A1C, and leptin,^21^^,^^22^ along with a proxy for pack-years. This composite structure distinguishes it from PhenoAge, another second-generation clock which similarly incorporates surrogate biomarkers including C-reactive protein, but does not include pack-years. Previous studies have shown that GrimAge correlates more strongly with both direct and indirect smoking exposures, such as parental smoking history, than other clocks,^14^^,^^23^ raising the question of whether its prognostic value in lung cancer primarily reflects cumulative tobacco exposure.

Our sensitivity analyses indicate otherwise. Self-reported pack-years explained only 9.6% of variance in GrimAge2 EAA and adjustment for pack-years minimally attenuated the GrimAge2 association, while GrimAge2 enhanced model discrimination beyond pack-years alone. Consistent with Lu et al., who found that the DNAm-based surrogate outperformed self-reported pack-years in predicting mortality,^21^ the remaining variance likely reflects both superior measurement by avoiding recall bias as well as individual biological variability that cumulative exposure alone cannot capture. Notably, GrimAge is also unique from other clocks in that it includes a surrogate for TIMP-1, which is secreted by lung tumors and has been identified as a predictive marker of poor prognosis in lung cancer,^24^ and has been associated with other lung diseases such as chronic obstructive pulmonary disease (COPD) and pulmonary fibrosis.^25^ The ability of GrimAge2 to incorporate smoking-related information while providing prognostic value beyond self-reported metrics reinforces the biological relevance of GrimAge2 in lung cancer prognosis, where smoking remains the dominant risk factor and modulator of disease progression.^26^^,^^27^^,^^28^

Our findings add nuance to this understanding by suggesting that the impact of EAA on mortality risk is stratified by smoking status. While prior research has focused on the effect of smoking on accelerating aging, including persistent DNA methylation changes in ever-smokers,^14^^,^^29^ our findings indicate that smoking status confers additional information on risk beyond that captured by EAA. Specifically, current smokers exhibited substantially higher mortality risk at comparable levels of GrimAge2 EAA than either former or never smokers. While current smokers in our sample had the highest mean GrimAge2 EAA, both highly and moderately accelerated groups contained comparable numbers of current and former smokers, indicating that the observed differences in mortality are unlikely to be solely driven by underlying differences in EAA. Additionally, in both the primary RSF model and sensitivity analyses, smoking status consistently ranked second in variable importance after GrimAge2 EAA and showed variable importance values of similar magnitude, indicating that both features contribute meaningfully to survival prediction.

This interpretation is further supported by prior research showing that continued smoking after cancer diagnosis is broadly associated with adverse clinical outcomes, including faster disease progression, poorer treatment response, and increased overall mortality.^30^ Given the known systemic effects of smoking, including immune system dysregulation and chronic inflammation,^31^^,^^32^ it is plausible that ongoing tobacco exposure may interact with the biological pathways that are captured by the GrimAge2 clock in ways not fully reflected by EAA alone. Although our data reflect smoking status only at the time of diagnosis, these findings highlight the need for personalized prognostic models in NSCLC that account for both smoking history and epigenetic aging dynamics.

This pattern parallels that of another aging-related prognostic biomarker of survival in early-stage lung cancer, known as clonal hematopoiesis of indeterminate potential (CHIP).^33^^,^^34^ CHIP arises from the accumulation of somatic mutations in hematopoietic stem cells and has been linked to systemic inflammation and accelerated epigenetic aging.^35^^,^^36^ A 2021 study by Nachun et al. found that individuals who had CHIP as well as positive EAA in both Hannum and GrimAge clocks had greater mortality than those with only CHIP or positive EAA alone, illustrating how aging-associated predictors that share overlapping biological pathways may still capture distinct dimensions of risk, such that relying on EAA alone may underestimate the full spectrum of aging-related mortality risk.^36^

The observed non-linear relationship between EAA and mortality is also noteworthy. In our models, risk appeared to plateau above EAA values of +5 years and below EAA values of −6 years. One possible interpretation is that, beyond these thresholds, other factors such as chronological age, tumor biology, or DNA repair capacity may play a more dominant role in determining survival, reducing the incremental prognostic value of EAA. However, these patterns reflect modeled associations, rather than causality, and should be viewed as hypothesis-generating observations rather than definitive biological cutoffs. If validated in larger cohorts, such thresholds could eventually help define ranges of EAA associated with elevated or reduced mortality, potentially informing risk stratification or intervention strategies.

### Limitations of the study

Several limitations warrant further consideration. First, our extreme phenotype sampling design, while enabling power in detecting differences associated with survival, produces optimistic estimates of model discrimination. We applied IPSW to approximate population-level associations among extreme survivors in the eligible Boston Lung Cancer Study (BLSC) cohort; however, because intermediate survivors were excluded from sampling, our models do not reflect discrimination across the full survival spectrum. We therefore emphasize relative changes in model performance rather than absolute discrimination metrics, and the need to confirm generalizability in cohorts with a more complete survival distribution.

Second, our modest sample size limited both multivariable adjustment and predictive modeling. While our primary analyses used a parsimonious covariate set, clinical sensitivity analyses included multiple parameters to adjust for stage, histology, and adjuvant therapy, and should therefore be interpreted as checks on robustness rather than definitive fully adjusted estimates. Moreover, the sample size precluded stratification by histology to examine whether EAA associations differ across lung cancer subtypes, which may indicate distinct biological relationships. The small sample size also increases the risk of overfitting, particularly for flexible machine learning methods such as random forests, and may lead to optimistic estimates despite our use of out-of-bag error and repeated fitting.

Third, we also lacked systematic data on performance status, comorbidities, and details on treatment, which influence survival and may act as confounders if these factors affect EAA. Prior studies have linked GrimAge to performance measures such as grip strength,^37^ as well as to lung diseases such as COPD,^38^ possibly through its incorporation of relevant biomarkers like TIMP-1. This suggests EAA may capture aspects of clinical factors that could influence both treatment selection and survival outcomes in early-stage NSCLC; though whether EAA primarily reflects comorbidities themselves or underlying biological processes warrants further investigation.

Fourth, our sample was diagnosed between 1998 and 2008, preceding several developments in contemporary care of early-stage NSCLC such as widespread adoption of video and robotic assisted surgical techniques and advancements in targeted perioperative immunotherapies.^39^^,^^40^ Additionally, patients who were not treated by surgical resection were excluded from our sample, and no participants received immunotherapy as adjuvant therapy, which limit the generalizability of our results particularly if treatment choice corresponds to EAA. While surgical resection remains the standard of care for early-stage NSCLC, survival has improved over time due to multiple factors related to improved quality of treatment and care. Whether EAA retains prognostic value in contemporary treatment settings, particularly for modalities not represented in our sample, requires validation in recent cohorts.

Finally, our study population had limited representation of never smokers, a growing population particularly suited for the applications of epigenetic age as a biomarker for lung cancer prognosis,^41^ which further restricted our analysis of the differences between ever and never smokers with respect to EAA.

Future research should clarify the potential mechanisms through which epigenetic aging influences survival in lung cancer. Studies incorporating markers of inflammation, immune cell composition, and other systemic processes may help determine whether EAA represents an intermediary pathway linking tobacco exposure to cancer-related outcomes. Longitudinal analyses of epigenetic aging in relation to smoking status, including duration and timing of smoking cessation, could help determine whether improved survival in former smokers arises from mechanisms that influence epigenetic aging, and whether mortality risk associated with EAA can be modified through smoking cessation.

Together, our findings support the potential utility of blood-based GrimAge2 as a readily accessible and clinically relevant biomarker that improves survival prediction in early-stage NSCLC beyond traditional risk factors. While validation in larger, contemporary cohorts is needed, these results highlight the potential of epigenetic aging measures in personalizing cancer prognostication.

## Resource availability

### Lead contact

Further information and requests for resources should be directed to and will be fulfilled by the lead contact, David C. Christiani (dchris@hsph.harvard.edu).

### Materials availability

This study did not generate unique reagents.

### Data and code availability

• Raw IDAT files used to generate epigenetic age have been deposited at GEO:GSE347344 and are publicly available as of the date of publication. De-identified individual-level patient data supporting the results reported in this study have been deposited at Zenodo and are publicly available as of the date of publication at https://doi.org/10.5281/zenodo.21689133. Summary statistics derived from survival analyses reported in this study have been also been deposited at Zenodo at https://doi.org/10.5281/zenodo.21676287.
• All original code has been deposited at Zenodo and is publicly available as of the date of publication at https://doi.org/10.5281/zenodo.21622402.
• Any additional information required to reanalyze the data reported in this study is available from the lead contact upon request.

## Acknowledgments

Supported by 10.13039/100000002NIH (10.13039/100000054NCI) grants U01CA209414, R01CA249096, and R01CA269398.

## Author contributions

I.G., M.D., and D.C.C. conceived of the present study. I.G. primarily analyzed and interpreted the data, with additional interpretation contributed by M.D., Y.L., M.L., and J.F.G.; L.S. performed laboratory analyses, and J.K. managed and processed sample data. M.L., J.F.G., and D.C.C. contributed to original study design, M.L. additionally contributed to patient recruitment of the study, and D.C.C. served as the principal investigator of the study. I.G. and D.C.C. verified the underlying data and drafted the manuscript, and Y.L. assisted with statistical analysis and editing. All authors reviewed and approved the final manuscript.

## Declaration of interests

J.F.G. has served as a compensated consultant for Amgen, AstraZeneca, Daiichi Sankyo, Mariana Therapeutics, Mirati Therapeutics, Merus Pharmaceuticals, Nuvalent, Pfizer, Novocure, AI Proteins, Novartis, Silverback Therapeutics, Sanofi, Summit Therapeutics, Bristol Myers Squibb, Genentech, ITeos Therapeutics, Revolution Medicine, Merck, Moderna Therapeutics, Takeda; has received honorarium from Novartis, Merck, and Pfizer, received institutional research funding from Adaptimmune, Alexo Therapeutics, AstraZeneca, Blueprint Medicines, Bristol Myers Squibb, Genentech, Merck, Moderna Therapeutics, Novartis, NextPoint Therapeutics, Palleon Pharmaceuticals; research support from Novartis, Genentech and Takeda, grant support from Moderna Therapeutics, and has equity in AI Proteins; and has an immediate family member who has equity in Ironwood Pharmaceuticals and Alkeus Pharmaceuticals.

## STAR★Methods

### Key resources table

**Table:** 

REAGENT or RESOURCE	SOURCE	IDENTIFIER
**Biological samples**		

Peripheral blood samples	Boston Lung Cancer Study (BLCS), MGH & DCFI	IRB protocol number: 1999P004935

**Critical commercial assays**		

Illumina Infinium MethylationEPIC BeadChip (850K)	Illumina	

**Deposited data**		

Raw methylation data	This paper	GEO:GSE347344
Analyzed data	This paper	https://doi.org/10.5281/zenodo.21676287, https://doi.org/10.5281/zenodo.21689133

**Software and algorithms**		

Clock Foundation DNAm Age Calculator	Clock Foundation	https://dnamage.clockfoundation.org
mice (v.3.19.0)	CRAN	RRID:SCR_02636; https://CRAN.R-project.org/package=mice
minfi	Bioconductor	RRID:SCR_012830; https://bioconductor.org/packages/minfi/
survey (v4.5)	CRAN	https://CRAN.R-project.org/package=survey
survival (v3.8-6)	CRAN	RRID:SCR_021137; https://CRAN.R-project.org/package=survival
survivalROC (v1.0.3.1)	CRAN	RRID:SCR_024415; https://cran.r-project.org/package=survivalROC
randomForestSRC (v3.6.2)	CRAN	RRID:SCR_026793; https://CRAN.R-project.org/package=randomForestSRC
Data analysis code	This paper; Zenodo	https://doi.org/10.5281/zenodo.21622402

### Experimental model and study participant details

#### Study population and sample collection

Samples were collected from participants in the Boston Lung Cancer Study (BLCS) is an ongoing cohort study of lung cancer patients enrolled at Massachusetts General Hospital and Dana-Farber Cancer Institute since 1992. By October 30, 2017, 1,755 early-stage non-small cell lung cancer (NSCLC) patients who underwent surgical resection with curative intent had been recruited with comprehensive clinical information including diagnosis date, sex, smoking history, cancer stage, and vital status.

Peripheral blood and tumor tissue were collected from newly diagnosed, early-stage NSCLC patients enrolled in BLCS under an established protocol at Massachusetts General Hospital and Dana-Farber Cancer Institute (for further detail, see prior descriptions^42^^,^^43^). The Massachusetts General Brigham (MGB) Health Care System Institutional Review Board (IRB) approved the study (IRB protocol number: 1999P004935). Written informed consent was obtained for all patients in the study. For participants included in the present analysis, blood samples used for DNA methylation profiling were obtained at the time of clinical management around diagnosis and surgical resection, which took place within 4 months of diagnosis.

#### Extreme phenotype selection

Among BLCS participants, the median survival time for patients who died of stage I NSCLC was 50.27 months (interquartile range [IQR]: 25.60–93.70) based on data available as of October 30, 2017. We defined short-term survivors as stage I patients who died within the median survival time, and long-term survivors as those who either died after >72 months or remained alive with >120 months of follow-up. We randomly selected 39 short-term survivors and 38 long-term survivors for DNA methylation profiling.

##### Study sample and follow-up

Of 77 patients with blood and tumor DNA methylation data, one failed array quality control and one was excluded after tumor sequencing revealed carcinoid histology, yielding a final analytic sample of 75 early-stage NSCLC patients diagnosed between 1998 and 2008. Survival data was continuously updated with extended follow-up through August 2023 (median follow-up time: 34.1 months [IQR: 19.5–186.2]). The final analytic sample had a mean age of 69.4 years (SD=8.2) and consisted of 43 male participants (57.3%) and 32 female participants (42.7%). Sex was not significantly associated with overall survival after adjustment for epigenetic age, with the exception of the Horvath EAA model (*p*=0.04). Additionally, 71 participants identified their race as White (94.7%), while the remaining 4 participants (5.3%) identified as Black; no participants identified as Hispanic or Latino.

### Method details

#### Calculation of DNA methylation-based age

DNA methylation was assayed using the 850K Illumina Infinium MethylationEPIC BeadChip. Blood samples were selected for analysis because selected clocks were developed and validated primarily in blood as measures of systemic aging. Raw IDAT files were submitted to the Clock Foundation DNA Methylation Age Calculator,^44^ where they were preprocessed and normalized using the minfi^45^ package and noob normalization. Estimates for Horvath,^44^ Hannum,^46^ GrimAge,^21^ PhenoAge,^47^ and GrimAge2^22^ were obtained, and epigenetic age acceleration (EAA) of each clock was defined as the residual of epigenetic age regressed onto chronological age, in line with previous studies.^48^^,^^49^^,^^50^^,^^51^^,^^52^ Due to high correlation between the GrimAge and GrimAge2 clocks (Pearson r=0.99), only GrimAge2 was retained for analysis to reduce redundancy. GrimAge2 was selected over GrimAge based on previous findings showing marginally improved prediction of morbidity and mortality.^53^

#### Imputation of missing data

For two participants with missing pack-years data, values were imputed using predictive mean matching with the R package mice^54^ with age at diagnosis, sex, stage, histologic subtype, and smoking status as predictors.

### Quantification and statistical analyses

All analyses were conducted in R (version 4.5.1) on a total sample size of *N*=75 participants; subgroup analyses with sample sizes are detailed in Results. Summary statistics are reported as mean (SD) for approximately normally distributed variables and median (IQR) otherwise; categorical variables are reported as n (%), where n denotes the number of participants. For descriptive comparison of baseline characteristics between survival strata in Table 1, approximately normal continuous variables were evaluated using two-sample t-tests and non-normal variables using Wilcoxon rank sum tests; categorical variables were assessed using Fisher’s exact tests. Two-sided *p*-values <0.05 were considered statistically significant for Cox models. Hazard ratios (HRs) are reported with 95% confidence intervals (CIs). Statistical details for each analysis are provided in Results, figure legends, and corresponding tables.

#### Inverse probability of selection weighting

To account for uneven sampling fractions of short- and long-term survivors in extreme phenotype sampling and approximate population-level associations among extreme survivors in the original cohort, we calculated inverse probability of selection weights (IPSWs).^55^^,^^56^ We defined weights as the inverse sampling fraction within each survival stratum based on eligibility status as of October 30, 2017. Specifically, we used the formula w_i_ = N_Ai_/n_Ai_, where A_i_ denotes the survival stratum, N_Ai_ is the number of eligible individuals per stratum, and n_Ai,_ is the number sampled. Among early-stage NSCLC patients who underwent surgery, 920 met criteria for extreme survival (369 short-term, 551 long-term). For short-term survivors, weights were calculated as w_s_ = N_s_/n_s_, with N_s_ = 369 and n_s_ = 39. For long-term survivors, weights were w_l_ = N_l_/n_l_, where N_l_ = 551 and n_l_ = 38. After exclusion of two participants as described above, the sample represented a pseudopopulation of 891 extreme survivors (369 short-term, 522 long-term). IPSW was used for Cox models and Kaplan-Meier survival curves using the survey package^57^ and incorporated into random survival forests with the case.wt parameter. Because sampling was based on survival outcomes, subsequent analyses were interpreted as estimating associations within the extreme survivor population rather than population-level survival functions.

#### Cox modeling of overall survival

Cox proportional hazards models were constructed using the survival^58^ and survey^57^ packages in R to estimate associations with mortality after cancer diagnosis. Overall survival was used as the primary endpoint in analyses due to the clinical relevance of all-cause mortality and incompleteness of cause-of-death classification; cause-of-death data is detailed in Table S1. The primary model included chronological age at diagnosis, sex, and smoking status as baseline covariates, which are all established predictors of mortality in lung cancer,^59^^,^^60^ with each EAA measure added as a continuous predictor. All models were IPSW-adjusted to account for selection bias.

Sensitivity analyses examined robustness to pack-years, clinical adjustment for cancer stage (IA, IB, I) and histology (adenocarcinoma [LUAD], squamous cell carcinoma [LUSC], Adenocarcinoma *in situ* [previously bronchioalveolar carcinoma or BAC], other), and effect modification of EAA with smoking status. To compare nested models for interaction, the Rao-Scott likelihood ratio test was applied to compare IPSW-adjusted models.

##### Model discrimination

For Cox models, Harrell’s C-index was used to compare models. For both Cox and RSF models, time-dependent AUC was computed using the survivalROC package^61^ at 6-month increments across follow-up. Time-dependent AUC was calculated in the analytic sample using risk scores from IPSW-weighted fitted models.

#### Random survival forest modeling

RSF models were fit in R with the randomForestSRC package^62^ using the same covariates as Cox models. To stabilize predictions and minimize overfitting, we grew 1,000 trees with a minimum node size of 10 and considered m=1 candidate predictor at each split to promote model diversity while maintaining stability with limited sample size. Inverse probability of selection weighting was applied using the case.wt parameter, which determines the probability of selection in bootstrap sampling. Out-of-bag (OOB) predictions are based on observations excluded from each tree’s bootstrap sample. To obtain robust estimates of predictive performance and account for variability between random forests, we re-fit each RSF model 500 times and computed the mean and standard deviation of time-dependent AUC, OOB error rates, Breiman-Cutler Variable Importance (BCVI), and predicted mortality risk of OOB observations for partial dependence plots.
