# Supplementary material for: Epigenetic age acceleration enhances prediction of overall survival in early-stage non-small cell lung cancer
Source: iScience. 2026 Oct 16;29(11):117843. doi: 10.1016/j.isci.2026.117843 (PMC13691256; doi:10.1016/j.isci.2026.117843)
Supplement: Supplementary file 1 [file mmc1.pdf]

iScience, Volume *29*

Supplemental information

Epigenetic age acceleration enhances prediction of overall survival in early-stage non-small cell lung cancer

Isabella Gandara, Mulong Du, Yi Li, Li Su, Jui Kothari, Michael Lanuti, Justin F. Gainor, and David C. Christiani

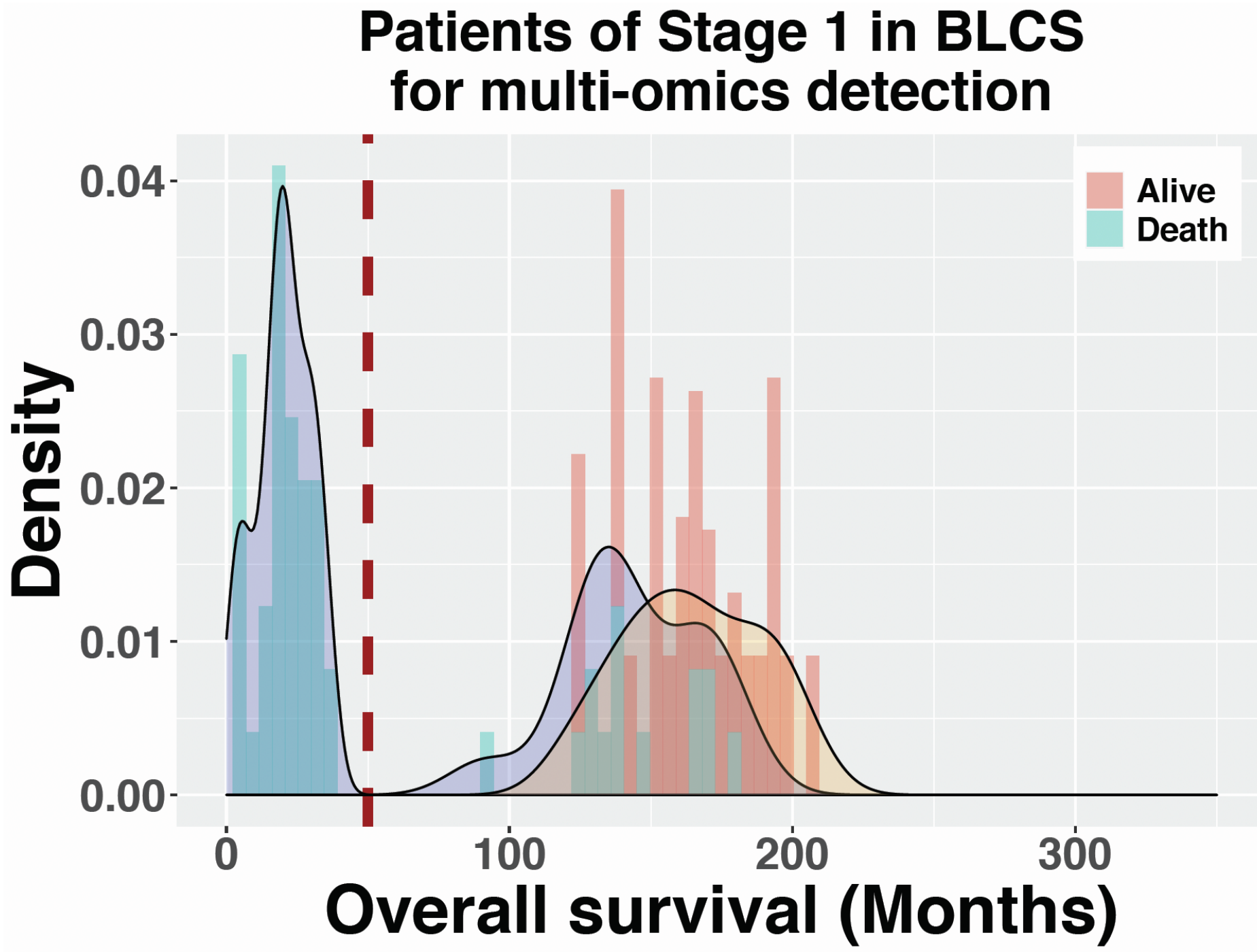

Figure S1. Distribution of overall survival in extreme survival sample. Short- and long-term survivors were selected from BLCS as described in Methods for multi-omic profiling in October 2017. Colors correspond to survival status of participants as of selection, with associated density plots overlaid. The dotted line represents the cutoff for short-term survival, defined by the median of the cohort (50.27 months).

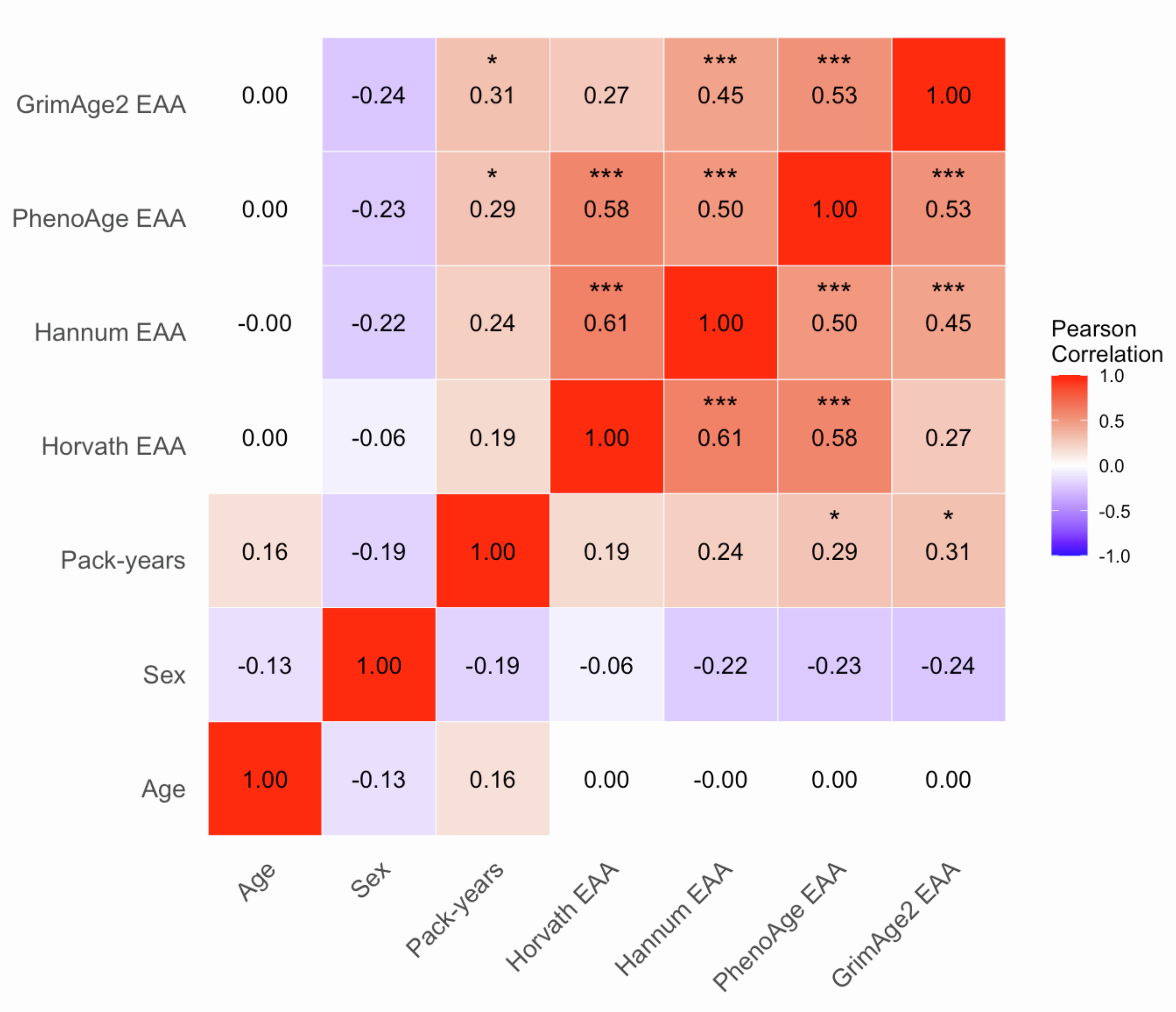

Figure S2. Correlation between EAA and continuous predictors. Asterisks denote correlations significant after Benjamini-Hochberg correction across all pairwise tests (* p <0.05; ** p<.01; *** p<0.001)

Table S1. Detailed Cause of Death Information

	**Overall** (N=75)	**Short-term** (n=39)	**Long-term** (n=36)
Deaths	63	39	24
**Cause of Death, n (%)**			
Lung cancer	26 (41.3%)	21 (53.8%)	5 (20.8%)
Other cause			
Secondary Cancer	4 (6.3%)	1 (2.6%)	3 (12.5%)
Treatment-related	1 (1.6%)	1 (2.6%)	0
Unknown/unspecified	30 (47.6%)	16 (41.0%)	14 (58.3%)
Missing cause	2 (3.2%)	0	2 (8.3%)

Table S2. Sensitivity Analysis: Pack-years and Clinical Factor Adjustment

		Cox					RSF		
**Adjustment Set**	**Clock**	**HR (95% CI)**	**p**	**ΔHR[a]**	**C-index**	**ΔC[b]**	**RSF Error**	**ΔError[b]**	**BCVI**
**Pack-years**	Base pack-years	–	–	–	0.70 (0.04)	–	37.9% (0.4)	–	–
	Horvath EAA	1.06 (1.01–1.10)	0.017	+0.1%	0.71 (0.04)	+0.009	37.1% (0.5)	−0.8	1.1% (0.2)
	Hannum EAA	1.08 (1.04–1.12)	<0.001	−1.0%	0.72 (0.04)	+0.016	36.4% (0.4)	−1.5	1.4% (0.2)
	PhenoAge EAA	1.05 (1.01–1.08)	0.004	−0.8%	0.71 (0.04)	+0.011	36.4% (0.4)	−1.5	2.3% (0.3)
	GrimAge2 EAA	1.06 (1.01–1.11)	0.023	−1.2%	0.73 (0.03)	+0.028	34.1% (0.4)	−3.8	4.7% (0.3)
**Clinical (Histology + Stage)**	Base clinical	–	–	–	0.72 (0.04)	–	35.7% (0.5)	–	–
	Horvath EAA	1.06 (1.00–1.12)	0.050	+0.4%	0.73 (0.04)	+0.012	34.6% (0.5)	−1.1	1.1% (0.2)
	Hannum EAA	1.09 (1.04–1.14)	<0.001	−0.2%	0.74 (0.04)	+0.023	34.0% (0.4)	−1.7	1.4% (0.2)
	PhenoAge EAA	1.06 (1.01–1.10)	0.009	+0.3%	0.74 (0.04)	+0.016	33.7% (0.5)	−2.0	2.4% (0.2)
	GrimAge2 EAA	1.07 (1.02–1.13)	0.007	+0.1%	0.74 (0.03)	+0.024	32.7% (0.5)	−3.1	4.3% (0.3)
**Clinical with Treatment (Histology + Stage + Adjuvant Therapy)**	Base clinical + treatment	–	–	–	0.72 (0.04)	–	36.5% (0.5)	–	–
	Horvath EAA	1.06 (1.00–1.12)	0.052	+0.3%	0.73 (0.04)	+0.014	35.7% (0.5)	−0.8	1.2% (0.2)
	Hannum EAA	1.09 (1.04–1.15)	<0.001	0%	0.74 (0.04)	+0.026	34.6% (0.4)	−1.9	1.3% (0.2)
	PhenoAge EAA	1.06 (1.02–1.11)	0.002	+0.8%	0.74 (0.04)	+0.019	34.5% (0.5)	−2.0	2.3% (0.2)
	GrimAge2 EAA	1.07 (1.02–1.13)	0.006	0%	0.75 (0.03)	+0.027	33.1% (0.5)	−3.4	4.3% (0.3)

[a] % change in EAA hazard ratio relative to primary model (age, sex, smoking, + EAA)
[b] Difference in predictive metrics (C-index for Cox, out-of-bag error rate for RSF) relative to base model within each adjustment set.

Cox proportional hazards and random survival model performance after adjustment for (1) pack-years, (2) clinical stage and histology and (3) clinical stage, histology, and treatment with adjuvant therapy following surgical resection. All models were IPSW-weighted and adjusted for age, sex, and smoking status.

Table S3. Detailed Kaplan Meier survival tables

Strata	0 years	5 years	10 years	15 years	20 years
Accelerated	419 (39)	145 (10)	145 (10)	58 (4)	29 (2)
Decelerated	472 (36)	377 (26)	362 (25)	218 (15)	145 (10)
High Acceleration	156 (16)	14 (1)	14 (1)	14 (1)	0 (0)
Low Acceleration	263 (23)	130 (9)	130 (9)	44 (3)	29 (2)
Low Deceleration	221 (17)	174 (12)	160 (11)	72 (5)	29 (2)
High Deceleration	250 (19)	203 (14)	203 (14)	145 (10)	116 (8)
Accelerated Current Smoker	195 (19)	44 (3)	44 (3)	0 (0)	0 (0)
Accelerated Former Smoker	224 (20)	102 (7)	102 (7)	58 (4)	29 (2)
Decelerated Current Smoker	57 (5)	29 (2)	29 (2)	14 (1)	0 (0)
Decelerated Former Smoker	347 (26)	290 (20)	276 (19)	160 (11)	102 (7)

Counts for IPSW-weighted Kaplan-Meier survival curves, corresponding to (in order from top to bottom) GrimAge2 acceleration status; benchmarked GrimAge2 acceleration status, as defined by partial dependence plots; and combined GrimAge2 acceleration and smoking status, with never smokers excluded due to low representation. Values outside parentheses are effective numbers at risk in the IPSW-adjusted pseudopopulation; unweighted numbers at risk from the analytic sample are shown in parentheses.

Table S4. Characteristics by GrimAge2 acceleration category

Characteristic	High Acceleration n=16	Low Acceleration n=23	Low Deceleration n=17	High Deceleration n=19
**Age, years**	71.3 (8.4)	66.7 (7.6)	71.4 (7.4)	69.4 (9.1)
**Male sex**	13 (81.3%)	11 (47.8%)	10 (58.8%)	9 (47.4%)
**Smoking status**				
Never smoker	0 (0%)	0 (0%)	1 (5.9%)	4 (21.1%)
Former Smoker	9 (56.3%)	11 (47.8%)	13 (76.5%)	13 (68.4%)
Current Smoker	7 (43.8%)	12 (52.2%)	3 (17.6%)	2 (10.5%)
**Pack-years**	57.5 (37.4–110.5)	45.4 (26.8–69.7)	42.2 (26.5–50.0)	28.9 (2.3–71.3)
**GrimAge2 EAA, years**	10.0 (3.8)	2.7 (1.5)	−2.8 (1.8)	−9.3 (2.7)
**Clinical Stage**				
1	0 (0%)	1 (4.3%)	0 (0%)	0 (0%)
1A	9 (56.3%)	11 (47.8%)	7 (41.2%)	11 (57.9%)
1B	7 (43.8%)	11 (47.8%)	10 (58.8%)	8 (42.1%)
**Received adjuvant therapy**	4 (25.0%)	1 (4.3%)	2 (11.8%)	1 (5.3%)
**3-year survival**	1 (6.3%)	9 (39.1%)	12 (70.6%)	14 (73.7%)
**Follow-up time, months**	16.1 (8.2–25.8)	28.9 (19.5–170.0)	128.3 (34.9–186.2)	195.1 (31.0–247.0)
**Time to death, months**	13.9 (6.9–22.7)	28.1 (19.5–133.9)	125.8 (34.3–168.9)	162.6 (21.5–195.1)

Table S5. Characteristics by GrimAge2 Acceleration and Smoking Status

	Accelerated (EAA > 0)		Decelerated (EAA < 0)		
**Characteristic**	**Current** (n=19)	**Former** (n=20)	**Current** (n=5)	**Former** (n=26)	**Never** (n=5)
**Age, years**	66.9 (8.8)	70.2 (7.4)	73.7 (8.5)	70.7 (7.9)	64.8 (9.0)
**Male sex**	12 (63.2%)	12 (60%)	2 (40%)	16 (61.5%)	1 (20%)
**Pack-years**	49.2 (36.1–69.7)	48.0 (26.3–87.3)	42.2 (37.3–42.3)	42.5 (22.5–72.5)	–
**GrimAge2 EAA, years**	5.8 (4.3)	5.7 (4.8)	−3.9 (4.2)	−6.0 (3.7)	−9.3 (4.2)
**Clinical Stage**					
1	0 (0%)	1 (5.0%)	0 (0%)	0 (0%)	0 (0%)
1A	9 (47.4%)	11 (55.0%)	4 (80.0%)	11 (42.3%)	3 (60.0%)
1B	10 (52.6%)	8 (40.0%)	1 (20.0%)	15 (57.7%)	2 (40.0%)
**Received adjuvant therapy**	1 (5.3%)	4 (20.0%)	0 (0%)	3 (11.5%)	0 (0%)
**3-year survival**	3 (15.8%)	7 (35.0%)	2 (40.0%)	20 (76.9%)	4 (80.0%)
**Follow-up time, months**	21.7 (16.5–28.9)	29.8 (15.3–170.2)	34.9 (27.6–135.8)	165.5 (91.5–243.3)	240.1 (151.7–247.0)
**Time to death, months**	21.7 (16.5–28.9)	21.1 (13.9–34.1)	34.9 (27.6–135.8)	128.3 (21.5–186.2)	151.7 (31.0–247.0)

Values are mean (SD), median (IQR), or n (%)
